# Prevalence and predictors of cervical cancer screening among HIV-positive women in rural western Uganda: insights from the health-belief model

**DOI:** 10.1186/s12885-023-11683-8

**Published:** 2023-12-08

**Authors:** Easwaran Vigneshwaran, Narayana Goruntla, Bhavana Reddy Bommireddy, Mohammad Jaffar Sadiq Mantargi, Bhavani Mopuri, Durga Prasad Thammisetty, Kasturi Vishwanathasetty Veerabhadrappa, Sarad Pawar Naik Bukke

**Affiliations:** 1 Department of Clinical Pharmacy, College of Pharmacy, King Khalid University, Abha, Kingdom of Saudi Arabia; 2 Department of Clinical Pharmacy and Pharmacy Practice, School of Pharmacy, Kampala International University, Western Campus, Kampala, Uganda; 3 Department of Pharmacy Practice, Raghavendra Institute of Pharmaceutical Education and Research (RIPER) – Autonomous, Anantapur, Andhra Pradesh India; 4 Department of Pharmaceutical Sciences, Pharmacy Program, Batterjee Medical College, 21442 Jeddah, Saudi Arabia; 5 Department of Pharmacy Practice, Sri Padmavathi School of Pharmacy, Vaishnavi Nagar, Andhra Pradesh, Tiruchanoor, Tirupati, 517503 India; 6 Department of Pharmacognosy and Pharmaceutical Chemistry, School of Pharmacy, Kampala International University, Western Campus, Kampala, Uganda; 7 Department of Pharmaceutics and Pharmaceutical Technology School of Pharmacy, Kampala International University, Western Campus, Kampala, Uganda

**Keywords:** Cervical cancer, Screening, HIV, Women, Health belief model, Uganda

## Abstract

**Background:**

Women living with HIV are at increased risk of developing cervical cancer (CC). Screening for cancer is an important preventive strategy for the early detection of precancerous lesions and its management. There has been inadequate evidence on cervical cancer screening (CCS) practices among HIV-positive women in rural western Uganda. This study aimed to assess the prevalence and predictors of CCS among HIV-positive women, as well as knowledge and practices regarding cervical cancer screening**.**

**Methods:**

A cross-sectional, analytical study was conducted among HIV-positive women attending HIV care facilities located in rural settings of western Uganda. A validated and interview-based data collection form was used to capture statistics regarding demographics, HIV care, obstetric profile, health belief constructs, and knowledge and history of CCS from the participants. Bivariate and multivariate logistic regression analyses were used to correlate women’s characteristics and health beliefs toward CCS practices.

**Results:**

The prevalence of CCS among HIV-positive women was found to be 39.1% (95%CI: 14.0–71.7). A multivariate logistic regression analysis showed that post-secondary education attainment (AOR = 3.21; 95%CI = 2.12–7.28), four years or more lapsing after being diagnosed as HIV-positive (AOR = 2.87; 95%CI = 1.34–6.13), having more than one child (AOR = 1.87; 95%CI = 1.04–3.35), antenatal care attendance (AOR = 1.74; 95%CI = 1.02–3.43), post-natal care attendance (AOR = 3.75; 95%CI = 1.68–5.89), and having good knowledge regarding CC (AOR = 1.26; 95%CI = 1.98–3.02) were positively associated with adherence to CCS among HIV-positive women in western Uganda. Health Belief Model (HBM) constructs like the perceived risk of developing CC (AOR = 1.82; 95%CI = 1.16–2.01), worries about developing CC (AOR = 5.01; 95%CI = 4.26–8.32), believing that CC leads to death (AOR = 2.56; 95%CI = 1.64–3.56), that screening assists in early identification (AOR = 2.12; 95%CI = 1.84–3.74) and treatment (AOR = 4.63; 95%CI = 2.78–6.43) of precancerous lesions, reducing the risk of mortality (AOR = 1.84; 95%CI = 1.12–2.75), and the reassurance provided by negative test results (AOR = 2.08; 95%CI = 1.33–4.22) were positively associated with adhering to CCS. A female doctor performing the screening (AOR = 2.02; 95%CI = 1.57–3.98) as well as offering a free screening service (AOR = 3.23; 95%CI = 1.99–4.38) were significantly associated with CCS. Meanwhile, screening being painful (AOR = 0.28; 95%CI = 0.12–0.45), expensive (AOR = 0.36; 95%CI = 0.24–0.53), time-consuming (AOR = 0.30; 95%CI = 0.19–0.41), embarrassing (AOR = 0.02; 95%CI = 0.01–0.06), and the fear of positive results (AOR = 0.04; 95%CI = 0.02–0.10) were found to have a significant negative association with adhering to CCS.

**Conclusions:**

Only one-third of HIV-positive women had undergone CCS. Variables including secondary education attainment, four years or more lapsing after being diagnosed as HIV-positive, having more than one child, antenatal care attendance, post-natal care attendance, and knowledge about CC were positively associated with CCS adherence. Educational programs should be geared towards the risk of CC, severity of cases, benefits of screening, and reducing barriers associated with screening, which can significantly improve cervical CCS among HIV-positive women. The study proposes the incorporation of free screening services and the inclusion of trained female staff in CC prevention policies to improve CCS.

## Introduction

Globally, cervical cancer ranks as the fourth-most prevalent cancer among women, resulting in 311,365 fatalities in 2018 [1]. Cervical cancer can be prevented through human papilloma virus (HPV) vaccination, early screening, and timely treatment of precancerous lesions. Cervical cancer-related mortality and morbidity predominantly affect low- and middle-income countries (LMICs) due to inadequate screening procedures and a deficient HPV vaccination system. This deficiency results in approximately 90% of the cases occurring in these regions [2]. Uganda has the seventh-highest incidence rate of cervical cancer globally, and the second-highest in East Africa, with a rate of 28.8 cases per 100,000 people per year [3].

Based on the statistics disclosed by the ICO/IARC HPV Information Center, it has been determined that cervical cancer ranks as the most prevalent form of cancer among women in Uganda [4]. In Uganda, the annual incidence of cervical cancer is reported to be 6959 cases, with a corresponding mortality rate of 4607 deaths. Multiple sexual partners, early age of first sexual activity, the use of hormonal contraceptives, smoking, HPV, and HIV infections are significant risk factors for cervical cancer [5]. HPV is the main cause of cervical cancer [4]. Cervical cancer is the primary cause of cancer-related fatalities in women in Uganda and sub-Saharan Africa. It is estimated that approximately 3.6% of women in the general population are affected by HPV 16 or/ and 18 infections. The HPV16/18 strains are associated with 57.0% of invasive cervical cancer cases [4]. Vaccination against these prevalent strains of HPV has the capacity to significantly decrease the occurrence of HPV-related illnesses, such as cervical and other anogenital cancers. HPV vaccines targeting HPV 16 and 18 have been accessible in Uganda since 2006 [6]. The initial HPV pilot vaccination was introduced in Uganda in 2008, specifically in the Nakasongola and Ibanda districts, with the aim of evaluating the practicality of the intervention. In 2012, it was subsequently implemented in 12 additional districts [7]. The successful implementation of these initial test projects cleared the path for a nationwide expansion of the HPV vaccination program in November 2015 [8]. The adoption of HPV vaccination is limited, as less than 50% of the intended girls receive their second subsequent dose. Several factors, including individual, community, and health system variables, influence the administration of HPV vaccination. To achieve high vaccination uptake in Uganda, it is important to adopt multifaceted approaches that target younger girls and empower both the girls themselves and their parents or carers to actively seek HPV vaccination services [9]. The 4-valent (4vHPV) vaccine is currently the EPI vaccine recommended; it is available, free of charge in Uganda, and protects against precancerous lesions and genital warts caused by the HPV6/11/16/18 types.

Women living with HIV are at a six-fold increased risk of developing cervical cancer compared to women without HIV [1]. The high prevalence of HIV (7.1%) among women aged between 15 and 49 years is a major risk factor for cervical cancer in Uganda [4]. This higher risk of cervical cancer among HIV-positive women is manifested in the acquisition of HPV infection, the fast progression to cancer, the low cure rate of precancerous lesions, and the high rate of relapse following treatment [10]. Globally, it is estimated that 5% of cervical cancer cases are attributed to HIV. Eighty-five percent of HIV-positive women with cervical cancer live in sub-Saharan Africa [1, 11]. There is a need to scale up the prevention and management strategies (HPV vaccination, screening, and treatment of precancerous and cancerous lesions) to reduce the burden of cervical cancer among HIV-positive women in African countries [12].

Annual screening is recommended for HIV-positive women due to their increased susceptibility to cervical cancer, as highlighted by the Centers for Disease Control and Prevention (CDC) and Ugandan HIV treatment guidelines [13]. In 2010, the government of Uganda launched its strategic plan for cervical cancer prevention and control under the Ministry of Health strategic plan for cervical cancer prevention and control, 2010–2014. This strategic plan focuses on addressing barriers to country-wide cervical cancer control. Priority areas include public education and advocacy, vaccination, screening, and treatment of cervical precancerous lesions. The primary goals of this strategic plan are as follows: by 2015, to ensure that 90% of the population in Uganda has been provided with information, education, and communication (IEC) resources regarding cervical cancer; to vaccinate 80% of eligible girls (aged 10–14) against HPV; and to conduct screening and treatment for precancerous lesions in 80% of eligible women (aged 25–49) [14]. The decline in cervical cancer screening can be attributed to several factors, including the considerable distance individuals must travel to access screening facilities, prolonged waiting periods, financial constraints that render the service unaffordable, and women’s dissatisfaction with the quality of screening services provided [15].

Under a comprehensive cancer control program, the Uganda Cancer Institute offers free cervical cancer screening for Ugandan women [16]. Although cervical cancer screening services are free in Uganda, but it is limited to public healthcare facilities [2, 17, 18]. Women residing in rural settings of Uganda are lack to access the facilities offering cervical cancer screening services. A community-based survey conducted in Central Uganda revealed that transport costs to the screening facility were relatively high ranging from Uganda shillings 3000 (equivalent to US$ 1.2) to Uganda shillings 30,000 (equivalent to US$12) with an average expenditure of Uganda shillings 13,000 (equivalent to US$ 5.2). [19]

Though there is an implementation of a simple and cost-effective strategy called the see-and-treat (visual inspection with acetic acid, diagnostic colposcopy, and cryotherapy) approach in Uganda, there is still a low prevalence of cervical cancer screening in both the general population and HIV-infected women [16]. In Uganda, there are very few studies regarding cervical cancer screening among HIV-positive women. According to a nationwide study in 2016, 30.3% of HIV-positive women had been screened at least once for cervical cancer [9]. One more study conducted in an urban HIV care clinic shows a 44.0% cervical cancer screening rate [16]. There was a dearth of evidence on cervical cancer screening among HIV-positive women in rural settings of western Uganda. This study aims to assess the prevalence of cervical cancer screening among HIV-positive women and explore the factors associated with screening practices, using a Health Belief Model approach.

## Materials and methods

### Study design and settings

**Fig. 1:**
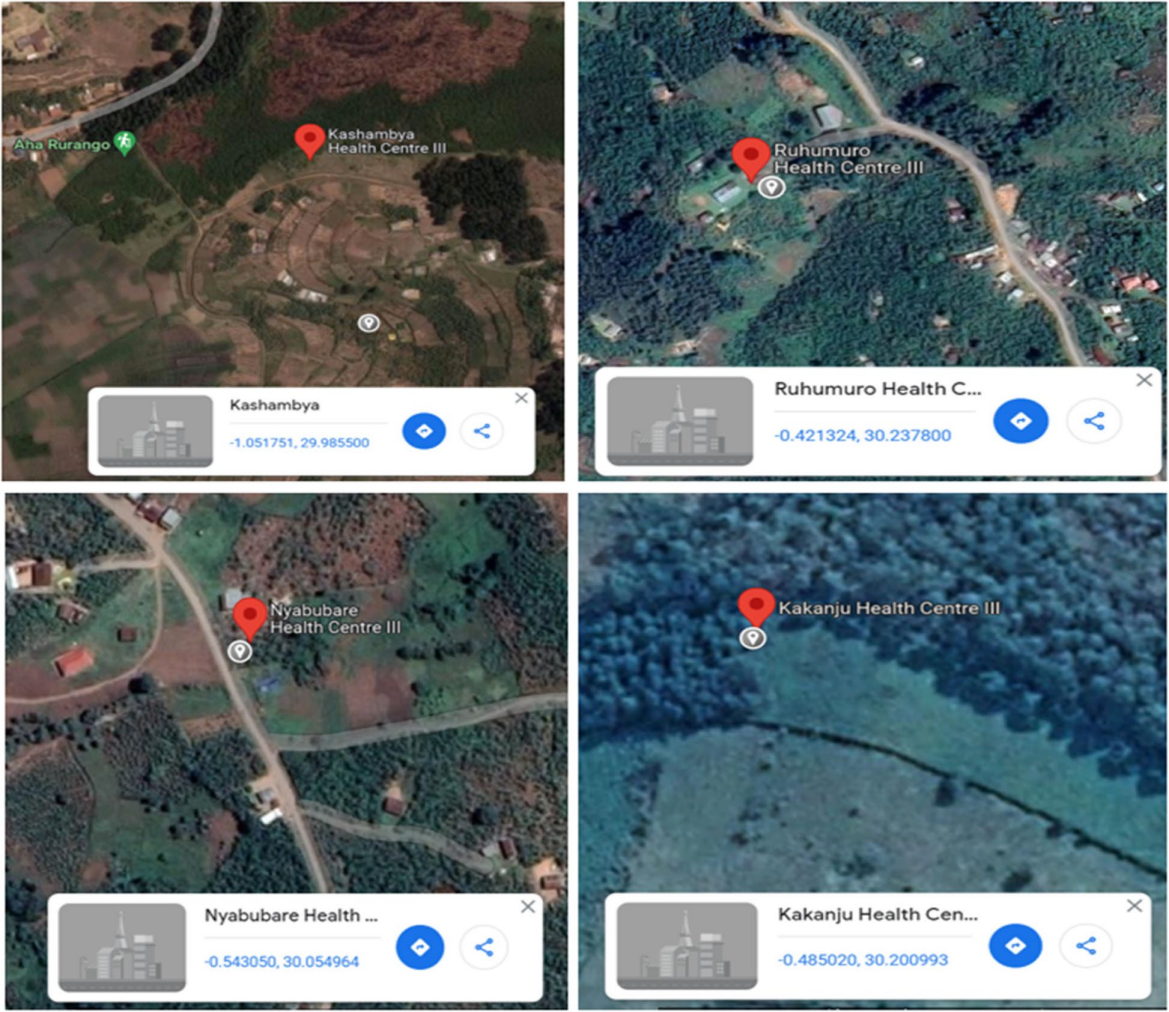
Google Maps of the study sites in Bushenyi district

An interview-based, cross-sectional, analytical study was conducted among HIV-positive women attending HIV care health centers located in rural settings of Bushenyi district, western Uganda. Bushenyi district has an area of 784 km^2^, and is located in the western region of Uganda; it has a population of 250,400 [20]. The Bushenyi population entirely depends on subsistence farming of matooke, cattle rearing, and coffee growing. The district is currently being served by a combined total of 13 public health centers and 5 private health centers, which are dedicated to meeting the healthcare requirements of HIV patients. Out of the total 18 health centers, 7 were situated in urban areas while the remaining 11 were situated in rural areas within Bushenyi district. The study was conducted in four health centers that were randomly chosen from rural areas in Bushenyi district. The selected study healthcare centers are Kashambya HC-III, Ruhumuro HC-III, Nyabubare HC-III, and Kakanju HC-III. The study centers' Google Maps are depicted in Fig. 1.

### Study population

The study included HIV-positive women who were willing to participate, and who were at least 18 years old. Women who were reported as critically ill, who had undergone hysterectomy with removal of the uterine cervix, and who were unable to respond to the questions were excluded from this study.

### Sample size determination

The sample size was calculated by using Epi-Info for Dos version 7.2.5.0 software (Center for Disease Control and Prevention—CDC, Atlanta, USA). The sample size was determined using the single proportional population formula called *n* = Zα/2^2^ *p*(1-p) / MOE^2^. By considering the 44% prevalence (p) of the cervical cancer screening rate among HIV-positive women, 80% power, Zα/22 of 1.96 at a 95% confidence interval, and a 5% margin of error (MOE), the sample size was calculated as 378 [13]. By assuming a 10% non-response rate, the final sample size was estimated to be 415. The study participants were recruited using a two-stage cluster sampling technique.

### Sampling procedure

In the initial stage, a total of four health centers (clusters) were selected by using a simple random sampling technique with a table of random number generators from the 11 health centers located in rural settings of western Uganda. Then, the sample size was evenly distributed to each health center (*n* = 104), and a systematic random sampling technique was used to capture the desired sample size from each cluster (ART clinic).

### Data collection tool

An interview-based data collection tool was prepared based on the current literature available on cervical cancer screening among HIV-positive women. The data collection tool comprises four components: I. Socio-demographics and clinical characteristics; II. Reproductive and obstetric characteristics; III. Awareness about cervical cancer and screening practices; and IV. Health Belief Model constructs towards cervical cancer screening.

### Socio-demographics and clinical characteristics

Socio-demographic information like age, marital status, and education of the participants were included in the study tool. The study participants were also queried regarding disclosure of HIV status to their sexual partners, HIV status of their partners, whether they are on anti-retroviral therapy (ART), their duration on ART, and their distances to health facilities in kilometers.

### Reproductive and obstetric characteristics

Each participant was asked to provide reproductive information, including age at first sexual intercourse, and history of any contraceptive use. Obstetrics data like the number of children, number of ante-natal visits, place of delivery, and post-natal check-ups were included in the data collection tool.

### Cervical cancer and screening practices

The data collection tool also comprises questions regarding knowledge about the need to screen cervical cancer, place of screening, likelihood of developing cervical cancer, source of information, and the affordability of screening. This section also focused on previous screenings for cervical cancer, numbers of times subjects underwent screening, and the last time subjects were screened.

### Health belief constructs

Participants’ perception about cervical cancer screening was analyzed using the Health Belief Model (HBM) hypothetical design [17]. The HBM constructs reflect the perceived susceptibility to develop cervical cancer among HIV-positive women (four items), perceived severity of cervical cancer among HIV-positive women (five items), perceived benefits of cervical cancer screening among HIV-positive women (two items), perceived barriers towards cervical cancer screening (five items), and cues to action (two items). Dichotomous responses (agree or disagree) were obtained for each item in this section. The distribution of the HBM constructs towards cervical cancer screening among HIV-positive women is represented in Table 3.

### Validation of data collection tool

An appropriately designed interviewer-based data collection form was prepared by including various components like socio-demographics, HIV status, reproductive information, obstetric profile, awareness and practices towards cervical cancer and its screening, and HBM constructs for cervical cancer screening (perceived susceptibility and severity of cervical cancer among HIV positive women, perceived benefits and barriers of cervical cancer screening, and cues to action). The data collection form was subjected to content validity and reliability testing.

The content present in the data collection form was evaluated by a panel of experts comprising an oncologist, obstetrics and genecology specialist (women), anthropologist, ART specialist, and a public health person. Expert opinions on the inclusion of a question/statement/component in the data collection tool were graded on a four-point Likert scale (strongly disagree = 1, disagree = 2, agree = 3, strongly agree = 4). The scale level content validity indicators like S-CVI/average number, S-CVI/Utility agreement, and item level content validity (I-CVI) were evaluated, and the content was adjusted to an acceptable margin (> 0.8) for each indicator.

The reliability of questions/statements/components indicated in the awareness about cervical cancer screening and HBM constructs of the survey tool were examined. The results of the reliability test performed in a pilot sample (*n* = 30) revealed a Cronbach’s alpha coefficient of 0.82 for awareness, 0.78 for susceptibility to cervical cancer, 0.80 for the severity of cervical cancer, 0.76 for benefits of cervical cancer screening, 0.80 for barriers to accept screening, and 0.78 for cue to action, representing acceptable internal consistency.

### Ethical considerations

The study protocol, data collection tools, and informed consent procedure was approved by Kampala International University—Research and Ethics Committee (KIU-REC 2023–014). The study was conducted in accordance with the provisions of the Declaration of Helsinki regarding research with human subjects. All of the eligible participants were approached by a female researcher who could speak the local language and English. The researcher explained the study protocol and objectives to the eligible participants, and received oral and written consent on a voluntary basis. For illiterate participants, informed consent was obtained from a legally authorized representative or local guardian. The participants were encouraged to share their thoughts and inquiries about the study openly, and received clarifications from the principal investigator. All of the participants had the complete right to withdraw from the study at any stage of the research process (consent, initiation, process, and completion). The study participants were not subjected to any harm, as the survey was conducted through interviews and did not involve any invasive procedures.

### Data collection

A total of 410 women who met the eligibility criteria were recruited in the study after clearing the informed consent process. A pre-tested, validated, interview-based data collection tool was used to obtain the information by a female researcher from the eligible participants in a private room. From each participant, information like socio-demographics, HIV status, reproductive information, obstetric profile, awareness and practices towards cervical cancer and its screening, and HBM constructs for cervical cancer screening were collected via a face-to-face interview using both open- and closed-ended questions. The interview was conducted for a period of 16–18 min to collect the survey details from each participant. The collected data were subjected to data analysis to estimate the prevalence and predictors of cervical cancer screening among HIV-positive women.

### Data analysis

To analyze the data collected from the women, IBM SPSS Statistics for Windows version 22.0 (IBM Corp., Armonk, NY, USA) was used. Before commencing the analysis, the data underwent cleaning, sorting, and processing within an Excel spreadsheet. The study employed bivariate and multivariate logistic regression analyses to examine the relationships between the independent variables (socio-demographics, clinical, reproductive, and obstetric characteristics of HIV-positive women) and the dependent variable (whether the women had ever been screened for cervical cancer). All significant variables with *p*-values less than 0.20 in the bivariate regression analysis were included in the multivariable model. A two-way *p*-value of less than 0.05 was considered statistically significant.

## Results

### Socio-demographics, clinical, reproductive, and obstetric profiles of study participants

Among 412 HIV-positive women, a total of 161 individuals were screened for cervical cancer, resulting in a prevalence rate of 39.1% (95% CI: 14.0–71.7). The mean (standard deviation) age of the participants involved in the study was 41.4 ± 11.3. Most of the participants were aged between 40 and 49 years (118; 28.8%), married (256; 62.4%), studied to a primary education level (239; 58.3%), and employed (232; 56.5%). The clinical profile of the participants revealed that the majority were suffering from other infectious diseases (348; 84.9%), disclosed their HIV status to their partners (386; 94.1%), were diagnosed between 2 and 4 years ago (136; 33.2%), and were located 15 km or farther from a health facility (128; 31.2%). The obstetric profile of the majority of HIV-positive women showed them attaining menarche at an age of less than 12 years (205; 50.0%); they had their first sexual intercourse at less than 16 years (223; 54.4%), used contraceptives (258; 62.9%), had a parity of one (156; 38.0%), visited an ANC at least once (259; 63.2%), and had a post-natal checkup (176; 42.9%). The distribution of the demographic, clinical, and obstetric profiles of the HIV-positive women are represented in Table 1.

**Table 1:** Socio-demographics, clinical, reproductive, and obstetric characteristics of HIV positive women (*n* = 412)

Variable	Frequency (%)
Age in Years (Mean ± SD)	41.4 ± 11.3
< 20	28 (6.8)
20–29	90 (22.0)
30–39	112 (27.3)
40–49	118 (28.8)
≥ 50	62 (15.1)
Marital status	
Married	256 (62.4)
Un-married	16 (3.9)
In relationship but not married	62 (15.1)
Divorced/Separated	47 (11.5)
Widowed	29 (7.1)
Education	
None	62 (15.1)
Primary	239 (58.3)
Secondary	88 (21.5)
Tertiary	15 (3.6)
University	6 (1.5)
Employment status	
Employed	232 (56.6)
Unemployed	178 (43.4)
Proximity to health facility (Km)	
< 5	124 (30.2)
5–9	93 (22.7)
10–14	65 (15.9)
≥ 15	128 (31.2)
Time since HIV/AIDS diagnosis in years (Mean ± SD)	2.48 ± 1.94
< 2	172 (41.9)
2–4	136 (33.2)
> 4	102 (24.9)
Disclosure of HIV status to partner	
Yes	386 (94.1)
No	24 (5.9)
Any other infectious disease	
Yes	348 (84.9)
No	62 (15.1)
Age at menarche in years (Mean ± SD)	12.78 ± 1.56
< 12	205 (50.0)
12–13	124 (30.2)
> 13	81 (19.8)
Age at first sexual intercourse (Mean ± SD)	16.58 ± 3.31
< 16	223 (54.4)
≥ 16	187 (45.6)
Contraceptive use	
Yes	258 (62.9)
No	152 (37.1)
Parity	
None	112 (27.3)
1	156 (38.1)
> 1	142 (34.6)
Visited ANC at least once	
Yes	259 (63.2)
No	151 (36.8)
Had postnatal check-up	
Yes	234 (57.1)
No	176 (42.9)

### Knowledge, affordability, and practices toward cervical cancer screening among study participants

The current study findings reveal that most HIV-positive women possess knowledge regarding cervical cancer (385; 93.9%) and the importance of screening (382; 93.2%), but less than half of them were aware of the facility where screening is conducted (169; 41.2%). Only 41.7% of the HIV-positive women could afford to pay for cervical cancer screening. The prevalence of practice of cervical cancer screening among HIV-positive women was found to be 39.3%. The distribution of the knowledge, affordability, and practices toward cervical cancer screening among HIV-positive women is represented in Table 2.

**Table 2:** Knowledge, affordability, and practices toward cervical cancer screening among HIV positive women (*n* = 410)

Knowledge	Frequency (%)
Knowledge about cervical cancer	
Yes	385 (93.9)
No	25 (6.1)
Knowledge about cervical cancer screening	
Yes	382 (93.2)
No	28 (6.8)
Knowledge of a cervical cancer screening facility	
Yes	169 (41.2)
No	241 (58.8)
Affordable to screen for cervical cancer	
Yes	171 (41.7)
No	239 (58.3)
Ever screened for cervical cancer	
Yes	161 (39.3)
No	249 (60.7)

### Health Belief Model (HBM) constructs of cervical cancer screening among HIV-positive women

According to the HBM, the constructs related to perceived susceptibility indicate that approximately half of the HIV-positive women were worried about CC (208; 50.7%), perceive a high risk of acquiring CC (212; 51.7%), and hold the belief that they may develop CC within a few years (194; 47.3%). A minority of HIV-positive women hold the perception that cervical cancer can result in serious health problems (178; 43.4%), necessitate hysterectomy (194; 47.3%), necessitate chemotherapy or radiotherapy (175; 42.7%), induce fear (196; 47.8%), jeopardize interpersonal relationships (188; 45.8%), and lead to mortality (180; 43.9%).

More than half of the HIV-positive women perceived barriers to CC screening; these included screening being painful (232; 56.6%), expensive (218; 53.2%), and a time-consuming procedure (229; 55.8%); they were afraid of being positive for the test (296; 72.2%), they were unaware of screening facilities (241; 58.8%), unaware about the frequency of screening (236; 57.6%), and unaware about the age of screening (232; 56.6%). Few HIV-positive women believe that screening helps to identify precancerous lesions (172; 41.9%), which are completely curable (168; 40.9%). Also, they believed that regular screening may reduce the worry (189; 46.1%) and deaths associated with CC (183; 44.6%). A preference for a female doctor for CC screening (195; 47.6%), and provision of screening services free of cost (192; 46.8%) are the identified cues among HIV-positive women. The distribution of HBM constructs of cervical cancer screening are represented in Table 3.

**Table 3:** Distribution of agree responses to HBM constructs of cervical cancer screening among HIV positive women (*n* = 410)

HBM constructs	Frequency (%)
Perceived susceptibility to get cervical cancer among HIV positive women	
Due to HIV, I worry a lot about developing cervical cancer	208 (50.7)
I am at high risk of developing cervical cancer than normal women	212 (51.7)
My chances of developing cervical cancer in the next few years are very high than normal women	194 (47.3)
Perceived severity/seriousness of cervical cancer among HIV positive women	
Cervical cancer is a serious health problem	178 (43.4)
Cervical cancer may lead to hysterectomy	194 (47.3)
Cervical cancer may lead to death	180 (43.9)
Cervical cancer makes women to opt either chemotherapy or radio therapy for cure	175 (42.7)
Thinking about cervical cancer frightens me	196 (47.8)
Cervical cancer threatens my relationship with others	188 (45.8)
Perceived barriers towards cervical cancer screening among HIV positive women	
Cervical cancer screening is painful	232 (56.6)
Cervical cancer screening is too expensive	218 (53.2)
Cervical cancer screening is time consuming procedure	229 (55.8)
It is too embarrassing to have a cervical cancer screening	284 (69.3)
I am afraid of positive for cervical cancer screening test	296 (72.2)
I am not aware of screening facility for cervical cancer	241 (58.8)
I am not aware of how often I need screening test	236 (57.6)
I am not aware at what age I can screen for cervical cancer	232 (56.6)
Perceived benefits of cervical cancer screening among HIV positive women	
Cervical screening test helps in identification of precancerous lesions	172 (41.9)
Precancerous lesions of cervix are completely curable	168 (40.9)
Regular cervical screening test will decrease my risk of dying due to cervical cancer	183 (44.6)
Regular cervical screening will reduce worry of cervical cancer if test is negative	189 (46.1)
Cues to action	
I prefer female doctor to perform cervical cancer screening test	195 (47.6)
I will only participate in screening if it is provided at no cost	192 (46.8)

### Determinants of cervical cancer screening

The findings of the logistic regression analysis revealed that HIV-positive women who studied to a level equal or above secondary school education [secondary (AOR = 3.21; 95% CI 2.12–7.28), tertiary (AOR = 9.82; 95% CI 2.58–42.12), university (AOR = 9.24; 95% CI 1.25–68.26)] were significantly associated with cervical cancer screening practices. Variables including time since diagnosis of HIV more than four years (AOR = 2.87; 95% CI 1.34–6.13), disclosure of HIV status (AOR = 2.46; 95% CI = 1.14–5.31), parity of more than one (AOR = 1.87; 95% CI 1.04–3.35), visited an ANC at least once (AOR = 1.74; 95% CI = 1.02–3.43), and visited a post-natal unit (AOR = 3.75; 95% CI = 1.68–5.89) were also significantly associated with cervical cancer screening practices among HIV-positive women. Knowledge regarding cervical cancer (AOR = 1.26; 95% CI = 1.18–3.02), screening (AOR = 1.28; 95% CI = 1.12–4.68), a facility that offers screening services (AOR = 18.06; 95% CI = 9.35–33.36), and patients who are able to afford screening for cervical cancer screening (AOR = 2.26; 95% CI = 1.28–4.15) were significantly associated with screening practices among HIV-positive women. The associations of participants’ demographics, clinical profile, obstetrics profile, and knowledge level towards cervical cancer screening practices among HIV-positive women are represented in Table 4.

**Table 4:** Logistic regression analysis of participant characteristics with cervical cancer screening among HIV positive women (*n* = 410)

Variable	Frequency (%)	Ever screened for CC	COR (95% CI),	*P-value*	AOR (95% CI)	*P-value*
		161				
Age in Years						
< 20	28 (6.8)	10 (35.7)	1.00	1.00	1.00	1.00
20–29	90 (22.0)	39 (43.3)	1.37 (0.57–3.42)	0.475		
30–39	112 (27.3)	45 (40.2)	1.21 (0.51–2.95)	0.665		
40–49	118 (28.8)	42 (35.6)	0.99 (0.42–2.35)	0.990		
≥ 50	62 (15.1)	25 (40.3)	1.21 (0.48–3.15)	0.678		
Marital status						
Married	256 (62.4)	101 (39.4)	1.00	1.00	1.00	1.00
Un-married	16 (3.9)	6 (37.5)	0.92 (0.30–2.62)	0.878		
In relationship but not married	62 (15.1)	24 (38.7)	0.97 (0.54–1.71)	0.914		
Divorced/Separated	47 (11.5)	19 (40.4)	1.04 (0.54–1.96)	0.900		
Widowed	29 (7.1)	11 (37.9)	0.94 (0.41–2.07)	0.874		
Education						
None	62 (15.1)	18 (29.0)	1.00	1.00	1.00	1.00
Primary	239 (58.3)	72 (30.1)	1.05 (0.57–1.98)	0.867	1.45 (0.71–2.93)	0.318
Secondary	88 (21.5)	54 (61.3)	3.84 (1.93–7.86)	< 0.001	3.21 (2.12–7.28)	0.001
Tertiary	15 (3.6)	12 (80.0)	9.46 (2.51–46.15)	< 0.001	9.82 (2.58–42.12)	< 0.001
University	6 (1.5)	5 (83.3)	11.75 (1.50–296.53)	0.007	9.24 (1.25–68.26)	0.002
Employment status						
Employed	232 (56.6)	92 (39.6)	0.99 (0.67–1.48)	0.855		
Unemployed	178 (43.4)	69 (38.8)	1.00	1.00	1.00	1.00
Proximity to health facility (Km)						
< 5	124 (30.2)	65 (52.4)	1.00	1.00	1.00	1.00
5–9	93 (22.7)	49 (52.7)	1.01 (0.59–1.74)	0.968	0.14 (0.05–1.22)	0.254
10–14	65 (15.9)	32 (49.2)	0.88 (0.48–1.61)	0.677	0.75 (0.38–1.72)	0.842
≥ 15	128 (31.2)	15 (11.7)	0.12 (0.06–0.23)	0.024	1.53 (0.83–2.82)	0.174
Time since HIV/AIDS diagnosis in years						
< 2	172 (41.9)	44 (25.6)	1.00	1.00	1.00	1.00
2–4	136 (33.2)	40 (29.4)	1.21 (0.73–2.01)	0.562	1.57 (0.77–3.20)	0.205
> 4	102 (24.9)	77 (75.5)	8.87 (5.08–15.86)	< 0.001	2.87 (1.34–6.13)	0.006
Disclosure of HIV status to partner						
Yes	386 (94.1)	157 (40.7)	3.42 (1.22–11.88)	0.019	2.46 (1.14–5.31)	0.023
No	24 (5.9)	4 (16.7)	1.00	1.00	1.00	1.00
Contraceptive use						
Yes	258 (62.9)	99 (38.4)	0.90 (0.60–1.36)	0.628		
No	152 (37.1)	62 (40.8)	1.00	1.00	1.00	1.00
Parity						
None	112 (27.3)	28 (25.0)	1.00	1.00	1.00	1.00
1	156 (38.1)	59 (37.8)	1.82 (1.07–3.14)	0.027	1.28 (0.64–2.17)	0.443
> 1	142 (34.6)	74 (52.1)	3.25 (1.90–5.63)	< 0.001	1.87 (1.04–3.35)	0.036
Visited ANC at least once						
Yes	259 (63.2)	132 (50.9)	4.36 (2.73–7.07)	< 0.001	1.74 (1.02–3.43)	0.036
No	151 (36.8)	29 (19.2)	1.00	1.00	1.00	1.00
Had postnatal check-up						
Yes	234 (57.1)	124 (52.9)	4.22 (2.72–6.63)	< 0.001	3.75 (1.68–5.89)	0.001
No	176 (42.9)	37 (21.0)	1.00	1.00	1.00	1.00
Knowledge about cervical cancer						
Yes	385 (93.9)	157 (40.8)	3.61 (1.22–10.73)	0.014	1.26 (1.18–3.02)	0.047
No	25 (6.1)	4 (16.0)	1.00	1.00	1.00	1.00
Knowledge about cervical cancer screening						
Yes	382 (93.2)	155 (40.6)	2.50 (1.02–6.31)	0.045	1.28 (1.12–4.68)	0.0328
No	28 (6.8)	6 (21.4)	1.00	1.00	1.00	1.00
Knowledge of a cervical cancer screening facility						
Yes	169 (41.2)	145 (85.8)	84.96 (43.64–165.39)	< 0.001	18.06 (9.35–33.36)	< 0.001
No	241 (58.8)	16 (6.6)	1.00	1.00	1.00	1.00
Affordable to screen for cervical cancer						
Yes	171 (41.7)	87 (50.9)	2.31 (1.53–3.47)	< 0.001	2.26 (1.28–4.15)	0.008
No	239 (58.3)	74 (30.9)	1.00	1.00	1.00	1.00

*Km*  Kilometer

### Association of HBM constructs towards cervical cancer screening

Within the framework of the perceived susceptibility, severity, and seriousness construct of the HBM, variables such as the perception of a high risk of developing cervical cancer (AOR = 1.82; 95% CI = 1.16–2.01), experiencing a lot of worry about developing cervical cancer (AOR = 5.01; 95% CI = 4.26–8.32), and cervical cancer could result in death (AOR = 2.56; 95% CI = 1.64–3.56) were significantly linked to the cervical cancer screening practices among HIV-positive women. Perceived barriers like screening being painful (AOR = 0.28; 95% CI = 0.12–0.45), expensive (AOR = 0.36; 95% CI = 0.24–0.53), time-consuming (AOR = 0.30; 95% CI = 0.19–0.41), embarrassing (AOR = 0.02; 95% CI = 0.01–0.06), and the fear of positivity (AOR = 0.04; 95% CI = 0.02–0.10) were significantly and negatively associated with cervical cancer screening practices among HIV-positive women. The perceived benefits of cervical cancer screening, such as the identification of precancerous lesions (AOR = 2.12; 95% CI = 1.84–3.74), early treatment of precancerous lesions (AOR = 4.63; 95% CI = 2.78–6.43) the reduction in mortality risk (AOR = 1.84; 95% CI = 1.12–2.75), and relieving participants’ concerns when the test results are negative (AOR = 2.08; 95% CI = 1.33–4.22), were found to be strongly associated with the practice of screening among women living with HIV.

Clues to action constructs like a female doctor performing screening (AOR = 2.02; 95% CI = 1.57–3.98) and offering a free screening service (AOR = 3.23; 95% CI = 1.99–4.38) were significantly associated with adherence to cervical cancer screening practices among HIV-positive women. Table 5 displays the results of the HBM indicators’ relationships with cervical cancer screening practices among HIV-positive women.

**Table 5:** Logistic regression analysis of HBM constructs with cervical cancer screening among HIV positive women (*n* = 410)

HBM constructs (Agreed)	Frequency (%)	Ever screened for CC	COR (95% CI)	*P-value*	AOR (95% CI)	*P-value*
***Perceived susceptibility to get cervical cancer among HIV positive women***						
Due to HIV, I worry a lot about developing cervical cancer						
Agree	208 (50.7)	126 (78.3)	7.29 (4.64–11.64)	< 0.001	5.01 (4.26–8.32)	< 0.001
Disagree	202 (49.3)	35 (21.7)	1.00	1.00	1.00	1.00
I am at high risk of developing cervical cancer than normal women						
Agree	212 (51.7)	99 (46.7)	1.92 (1.28–2.88)	0.001	1.82 (1.16–2.01)	0.042
Disagree	198 (48.3)	62 (31.3)	1.00	1.00	1.00	1.00
My chances of developing cervical cancer in the next few years are very high than normal women						
Agree	194 (47.3)	85 (43.8)	1.43 (0.96–2.14)	0.074	1.83 (0.48–1.68)	0.235
Disagree	216 (52.7)	76 (35.2)	1.00	1.00	1.00	1.00
***Perceived severity/seriousness of cervical cancer among HIV positive women***						
Cervical cancer is a serious health problem						
Agree	178 (43.4)	85 (47.7)	1.87 (1.25–2.81)	0.002	1.38 (0.29–1.52)	0.425
Disagree	232 (56.6)	76 (32.8)	1.00	1.00	1.00	1.00
Cervical cancer may lead to hysterectomy						
Agree	194 (47.3)	90 (46.4)	1.76 (1.18–2.64)	0.005	1.64 (0.12–1.56)	0.235
Disagree	216 (52.7)	71 (32.9)	1.00	1.00	1.00	1.00
Cervical cancer may lead to death						
Agree	180 (43.9)	94 (52.2)	2.65 (1.76–4.00)	< 0.001	2.56 (1.64–3.56)	0.035
Disagree	230 (56.1)	67 (29.1)	1.00	1.00	1.00	1.00
Cervical cancer makes women to opt either chemotherapy or radio therapy for cure						
Agree	175 (42.7)	78 (44.6)	1.47 (0.98–2.19)	0.057	0.26 (0.64–1.38)	0.122
Disagree	235 (57.3)	83 (35.3)	1.00	1.00	1.00	1.00
Thinking about cervical cancer frightens me						
Agree	196 (47.8)	80 (40.8)	1.13 (0.76–1.68)	0.539	-	-
Disagree	214 (52.2)	81 (37.8)	1.00	1.00	1.00	1.00
Cervical cancer threatens my relationship with others						
Agree	188 (45.8)	83 (44.1)	1.46 (0.98–2.17)	0.062	1.24 (0.84–1.98)	0.128
Disagree	222 (54.1)	78 (35.1)	1.00	1.00	1.00	1.00
***Perceived barriers towards cervical cancer screening among HIV positive women***						
Cervical cancer screening is painful						
Agree	232 (56.6)	69 (29.7)	0.39 (0.26–0.59)	< 0.001	0.28 (0.12–0.45)	0.003
Disagree	178 (43.4)	92 (51.7)	1.00	1.00	1.00	1.00
Cervical cancer screening is too expensive						
Agree	218 (53.2)	59 (27.1)	0.33 (0.22–0.49)	< 0.001	0.36 (0.24–0.53)	0.012
Disagree	192 (46.8)	102 (53.1)	1.00	1.00	1.00	1.00
Cervical cancer screening is time consuming procedure						
Agree	229 (55.8)	63 (27.5)	0.32 (0.21–0.48)	< 0.001	0.30 (0.19–0.41)	0.020
Disagree	181 (44.1)	98 (54.1)	1.00	1.00	1.00	1.00
It is too embarrassing to have a cervical cancer screening						
Agree	284 (69.3)	55 (19.4)	0.04 (0.02–0.08)	< 0.001	0.02 (0.01–0.06)	< 0.001
Disagree	126 (30.7)	106 (84.1)	1.00	1.00	1.00	1.00
I am afraid of positive for cervical cancer screening test						
Agree	296 (72.2)	66 (22.3)	0.05 (0.03–0.10)	< 0.001	0.04 (0.02–0.10)	< 0.001
Disagree	114 (27.8)	95 (83.3)	1.00	1.00	1.00	1.00
***Perceived benefits of cervical cancer screening among HIV positive women***						
Cervical screening test helps in identification of precancerous lesions						
Agree	172 (41.9)	95 (55.2)	3.20 (2.12–4.86)	< 0.001	2.12 (1.84–3.74)	0.024
Disagree	238 (58.0)	66 (27.7)	1.00	1.00	1.00	1.00
Precancerous lesions of cervix are completely curable						
Agree	168 (40.9)	106 (63.1)	5.78 (3.76–8.98)	< 0.001	4.63 (2.78–6.43)	< 0.001
Disagree	242 (59.0)	55 (22.7)	1.00	1.00	1.00	1.00
Regular cervical screening test will decrease my risk of dying due to cervical cancer						
Agree	183 (44.6)	85 (46.4)	1.83 (1.22–2.74)	0.003	1.84 (1.12–2.75)	0.042
Disagree	227 (55.4)	73 (32.1)	1.00	1.00	1.00	1.00
Regular cervical screening will reduce worry of cervical cancer if test is negative						
Agree	189 (46.1)	92 (48.7)	2.08 (1.39–3.13)	< 0.001	2.08 (1.33–4.22)	0.038
Disagree	221 (53.9)	69 (31.2)	1.00	1.00	1.00	1.00
***Cues to action***						
I prefer female doctor to perform cervical cancer screening test						
Agree	195 (47.6)	101 (51.8)	2.82 (1.88–4.26)	< 0.001	2.02 (1.57–3.98)	0.014
Disagree	215 (52.4)	60 (27.5)	1.00	1.00	1.00	1.00
I will only participate in screening if it is provided at no cost						
Agree	192 (46.8)	106 (55.2)	3.64 (2.40–5.55)	< 0.001	3.23 (1.99–4.38)	0.043
Disagree	218 (53.1)	55 (25.2)	1.00	1.00	1.00	1.00

## Discussion

HIV-positive women may experience an increased susceptibility to cervical cancer throughout their lifespan. The sequence of events begins with an elevated susceptibility to human papillomavirus (HPV) infection, followed by an accelerated transition from high-risk HPV infection to precancerous lesions, ultimately leading to the development of cervical cancer. Additionally, there is a diminished likelihood of regression of precancerous lesions and a heightened incidence of recurrence after treatment. The WHO advises that all HIV-positive women should have cervical cancer screening starting at age 25, and repeat the screening every 3 to 5 years [21]. This is the first study that was carried out in rural western Uganda to investigate the factors that influence cervical cancer screening in HIV-positive women.

### Prevalence of cervical cancer among HIV-positive women

The current study revealed that the prevalence of cervical cancer screening among HIV-positive women was found to be 39.1%. The prevalence of cervical cancer screening among HIV-positive women in rural western Uganda is comparatively lower when compared to studies conducted in high income countries (HICs) such as the United States (78.0%), Italy (91.0%), and Canada (82.0%) [22–24]. The reasons for this may include a robust healthcare system, proactive healthcare-seeking behavior, and the integration of cervical cancer screening and HIV care services in high income countries (HICs), which may facilitate access to information and screening services for HIV-positive women. A similar study conducted in urban Uganda also revealed a slightly higher prevalence (44.0%) than the current study [13]. This indicates that there is a need to strengthen the cervical cancer screening services in HIV centers located in both rural and urban areas of Uganda. Studies conducted in lower- and middle-income countries (LMICs) such as Ethiopia (10.0%), Nigeria (9.0%), and South Africa (32.0%) have reported a lower prevalence of cervical cancer screening compared to our study [25–27]. In Uganda and other LMICs, cervical cancer screening is provided as a standalone service. This results in HIV-positive women missing out on screening opportunities, despite their regular visits to HIV clinics for medication reviews and refills. The lower rate of cervical cancer screening among HIV-positive women may affect the timely detection, diagnosis, and management of precancerous lesions and cervical cancer. The present study reports a higher prevalence of cervical cancer screening compared to previous studies conducted in LMICs, which is potentially attributed to increased awareness regarding cervical cancer screening [25–27].

### Determinants of cervical cancer screening among HIV-positive women

The findings of the multivariate logistic regression analysis revealed that educational status, length of time since diagnosis, parity, ANC and PNC visits, disclosure of HIV status, and knowledge of HIV-positive women were significantly associated with adherence to cervical cancer screening practices. The study shows that educated HIV-positive women (secondary, tertiary, and university) were positively associated with cervical cancer screening practices compared to uneducated women. This indicates that educated women have better healthcare-seeking behaviors, awareness about screening, and decision-making power than uneducated women. Similar findings are also observed in studies conducted in Italy, Korea, Taiwan, and Ethiopia [23, 25, 28, 29]. Studies conducted in Laos and urban Uganda showed that education and cervical cancer practices are not significantly correlated among HIV-positive women [18, 30]. Though education brings awareness about cervical cancer and improves the decision power to undergo cervical cancer screening, HIV-positive women’s exposure to health facilities, information received from the healthcare professionals, and knowledge regarding cervical cancer play an important role in adhering to screening practices. This is the primary reason for the wide variations observed among different studies in relation to the influence of education on cervical cancer screening practices. This implies that regardless of the educational level of women, it is necessary to introduce educational interventions aimed at enhancing cervical cancer screening practices among HIV-positive women.

Women diagnosed with HIV four or more years ago, a parity of more than one, and attending ANC and PNC visits were more likely to undergo cervical screening. The primary reason for the practice of cervical cancer screening within this population is attributed to their regular and prolonged attendance at healthcare facilities for routine HIV care and treatment of HIV and other medical conditions. This facilitates the women in acquiring knowledge about cervical cancer and screening from healthcare professionals, which can enhance their cervical cancer screening practices. This is in line with the findings of the studies conducted in Kenya, northern Italy, and Ethiopia [23, 25, 31].

Women who had more than one child and regular attendance at ANC and PNC visits were more likely associated with undertaking cervical screening practices. This may be due to women who had children visiting healthcare facilities regularly for perinatal care in addition to routine HIV care follow-up compared to nulliparous women. Consequently, such individuals may have the opportunity to receive information and guidance on utilizing screening services from healthcare professionals. This finding is similar with the result of a study conducted in northwest Ethiopia [25].

Women who revealed their HIV status to their partner are significantly more likely to engage in cervical cancer screening practices compared to women who kept their HIV status hidden. This may be due to emotional support received from partners in caring for the HIV-positive women. A study conducted in central Uganda revealed that partners’ emotional support is strongly associated with cervical cancer screening among HIV-positive women [5]. Hence, it is imperative to promote and enhance knowledge regarding cervical cancer and its repercussions among men who will advocate for women’s screening practices.

It is crucial for HIV-positive women to possess adequate knowledge regarding cervical cancer, screening practices, and the availability of screening facilities. This knowledge is essential in promoting timely and appropriate uptake of screening, which aids in the detection of precancerous lesions and in the prevention of cervical cancer. In the current study, women’s knowledge level was significantly associated with their adherence to cervical cancer screening practices, similar with studies conducted in urban Uganda and Laos Republic [18, 30]. The results of this study suggest a need for the introduction of organized educational initiatives that specifically target women with inadequate practices regarding cervical cancer and screening. These programs aim to enhance their levels of knowledge, ultimately leading to an improvement in their adherence to cervical cancer screening practices. Women who reside far from healthcare facilities are poorly associated with cervical cancer screening practices. Longer geographical distances from healthcare facilities hinder women from attending regular visits for HIV, ART, ANC, and PNC. Consequently, these women also face challenges in accessing information about cervical cancer screening from healthcare professionals. This implies that community-based educational programs are essential for enhancing awareness of cervical cancer screening among women who have limited access to healthcare facilities, thereby benefiting the overall population.

### Health Belief Model constructs associated with cervical cancer screening among HIV-positive women

The findings of the multivariate logistic regression analysis revealed that Health Belief Model (HBM) constructs, such as perceived susceptibility of cervical cancer, severity and seriousness of cervical cancer, benefits of screening, and cues to action of screening, exhibited a significant association with their inclination to cervical cancer screening practices among HIV-positive women. Meanwhile, perceived barriers like screening are painful, expensive, time-consuming procedures, as well as embarrassing; fears of positivity concerns are significantly associated with lower odds of cervical cancer screening practices among HIV-positive women.

According to the perceived susceptibility construct, women who believe they might be at risk of developing CC and worry about getting CC are significantly associated with cervical cancer screening practices. This suggests that it is important to raise awareness among HIV-positive women about their increased susceptibility to developing cervical cancer in comparison to women without HIV. Ultimately, this has the potential to improve adherence to screening practices among HIV-positive women. While excessive worry about contracting cervical cancer can have a negative impact on women’s mental well-being, a reasonable level of concern is essential to motivate women to undergo cervical cancer screening. As with perceived susceptibility, the seriousness/severity of the CC can also play a vital role in the uptake of screening practices. Within the framework of the perceived seriousness construct, there was a significant correlation between HIV-positive women who believed that cervical cancer could lead to death and their adherence to screening practices. Previous studies conducted in LMICs and HICs showed a similar finding that perceived susceptibility and seriousness of cervical cancer predicted adherence to cervical cancer screening practices [32–36]. One potential reason for this result in our study could be that HIV-positive women who perceive the seriousness of the situation may possess knowledge about the comparable severity and fatality of cervical cancer.

The other variable that was positively associated with adhering to screening practices was the perceived benefits, such as screening helps in the early identification and treatment of precancerous lesions, reduces the risk of mortality, and provides reassurance for negative test results, which were positively associated with CCS. Cervical precancers are not cancers; they are lesions that may progress to cancer if left untreated. Screening tests such as the HPV test, the Papanicolaou (PAP) test, and visual inspection with acetic acid (VIA) can be utilized to identify precancerous lesions. HIV-positive women who possess knowledge about the efficacy of screening tests in detecting precancerous lesions at an early stage are more inclined to participate in screening procedures actively. Moreover, the timely identification of precancerous lesions on the cervix can be effectively treated through cryotherapy, a method that involves the eradication of abnormal cells through freezing, or LEEP, a technique that employs hot wire to destroy the abnormal cells. HIV-positive women should receive comprehensive counseling regarding the potential advantages of screening for timely identification and treatment of precancerous cervical lesions, which can effectively mitigate the risk of developing cervical cancer. The susceptibility findings indicate that women exhibit significant worry regarding the potential development of cervical cancer in the future. If the results indicate the absence of precancerous lesions, it can alleviate worry related to the likelihood of developing cervical cancer. The current study’s findings of the benefits offered by cervical cancer screening are in line with those of studies conducted in Botswana and Ethiopia [34, 35].

However, HBM barrier constructs such as screening being painful, expensive, time-consuming, embarrassing, as well as the fear of positive results were found to have a significant negative association with CCS practices among HIV-positive women. These findings suggest that there is a need to design educational interventions that prompt reductions in barriers and enhance knowledge about the benefits offered by cervical cancer screening for HIV-positive women.

The findings regarding the cues to actions construct reveal that a female doctor performing screening and offering free CC screening services are positively associated with cervical cancer screening. Involving female doctors encourages screening among participants, and can be correlated with the low screening rates seen among women who experienced embarrassment about CC screening. In order to overcome this barrier and improve the adherence to screening practices among HIV-positive women, it is necessary to only employ female personnel for conducting cervical cancer screenings. Cervical cancer screening facility access with no cost can encourage women to participate in the screening program.

The aforementioned findings illustrate the potential impact of HIV-positive women’s health beliefs on the cervical cancer screening decision-making process. The inclusion of findings from the HBM and the consideration of HIV-positive women’s characteristics that influence decision making can be taken into consideration when formulating cervical cancer screening policy and designing sensitization programs in HIV.

### Strengths and limitations

The scope of the study was restricted to HIV care clinics situated in rural regions of western Uganda. There is a need to expand this research in various regions of Uganda, to develop and offer a nation-wide cervical cancer screening program among HIV-positive women. The findings of this study help in addressing issues related to poor cervical cancer screening practices among HIV-positive women. The study did not endeavor to investigate the healthcare factors that are linked to the inadequate implementation of cervical cancer screening among HIV-positive women. The qualitative research focused on healthcare factors influencing cervical cancer screening among high-risk populations is necessary to strengthen screening uptake. Due to the cross-sectional nature of the study design, the actual causality of poor cervical cancer screening is not well established. The study participants were attending HIV care facilities; they were exposed to information on cervical cancer and screening availability, which can limit the generalization of screening practice findings to the general population of women. The primary strength of this study lies in its focus on rural western Uganda and its investigation of the factors related to cervical cancer screening practices among women who are HIV-positive. The utilization of interviews as the primary methodological approach in this study design allows for a reduction in response bias in the obtained findings.

## Conclusions

The study concludes that only one-third of HIV-positive women had undergone cervical cancer screening. Variables, including secondary education attainment, four years or more lapsing after being diagnosed HIV positive, having more than one child, antenatal care attendance, post-natal care attendance, and knowledge about cervical cancer, were positively associated with cervical cancer screening. Determinants of cervical cancer screening provide insights for public health interventions to prevent cervical cancer. These interventions may encompass activities such as advocating for the importance of perinatal visits, where women can acquire knowledge about CCS; creating visual materials for participants with no literacy or primary education levels; encouraging women in the early stages of HIV infection to screen for cervical cancer; and enhancing awareness regarding cervical cancer, screening, and healthcare facilities that offer screening services by providing structured educational programs. The educational programs should be geared towards the risk of CC, the severity of cases, the benefits of screening, and reducing barriers associated with screening can significantly improve cervical cancer screening among HIV-positive pregnant women. This study proposes the incorporation of free screening services and the inclusion of trained female staff in CC prevention policies to improve CC screening.

## Data Availability

The dataset used and/or analyzed during the current study are available from the corresponding author on reasonable request.

## Abbreviations

ANC: Antenatal care
AOR: Adjusted Odds Ratio
ART: Anti-Retroviral Therapy
CDC: Centers for Disease Control and Prevention
COR: Crude Odds Ratio
HBM: Health Belief Model
HIV: Human Immune Deficiency Virus
HPV: Human Papilloma Virus
I-CVI: Item level Content Validity Index
LMICs: Low- and Middle-Income Countries
PNC: Postnatal care
S-CVI: Scale level Content Validity Index
WHO: World Health Organization
